# Deep learning restores speech intelligibility in multi-talker interference for cochlear implant users

**DOI:** 10.1038/s41598-024-63675-8

**Published:** 2024-06-09

**Authors:** Agudemu Borjigin, Kostas Kokkinakis, Hari M. Bharadwaj, Joshua S. Stohl

**Affiliations:** 1 Weldon School of Biomedical Engineering, Purdue University, West Lafayette, 47907 IN USA; 2 Department of Speech, Language, and Hearing Sciences, Purdue University, West Lafayette, 47907 IN USA; 3 Waisman Center, University of Wisconsin-Madison, Madison, WI 53705 USA; 4 Concha Labs, San Francisco, CA 94114 USA; 5 Department of Communication Science and Disorders, University of Pittsburgh, Pittsburgh, PA 15213 USA; 6 North American Research Laboratory, MED-EL Corporation, Durham, NC 27713 USA

**Keywords:** Auditory system, Machine learning

## Abstract

Cochlear implants (CIs) do not offer the same level of effectiveness in noisy environments as in quiet settings. Current single-microphone noise reduction algorithms in hearing aids and CIs only remove predictable, stationary noise, and are ineffective against realistic, non-stationary noise such as multi-talker interference. Recent developments in deep neural network (DNN) algorithms have achieved noteworthy performance in speech enhancement and separation, especially in removing speech noise. However, more work is needed to investigate the potential of DNN algorithms in removing speech noise when tested with listeners fitted with CIs. Here, we implemented two DNN algorithms that are well suited for applications in speech audio processing: (1) recurrent neural network (RNN) and (2) SepFormer. The algorithms were trained with a customized dataset ($\sim$ 30 h), and then tested with thirteen CI listeners. Both RNN and SepFormer algorithms significantly improved CI listener’s speech intelligibility in noise without compromising the perceived quality of speech overall. These algorithms not only increased the intelligibility in stationary non-speech noise, but also introduced a substantial improvement in non-stationary noise, where conventional signal processing strategies fall short with little benefits. These results show the promise of using DNN algorithms as a solution for listening challenges in multi-talker noise interference.

## Introduction

Cochlear implant (CI) users typically achieve satisfactory speech intelligibility in quiet environments such as a sound booth used for testing in audiology clinics. However, they encounter difficulty hearing and comprehending speech in noisy social settings. There have been significant efforts towards the development of noise reduction algorithms in the field of hearing aids, and these algorithms are increasingly being translated into both CI research and commercial CI products. Noise reduction algorithms can be classified into single- vs multi-microphone strategies, as reviewed by^1,2^.

A single-microphone noise reduction strategy involves processing the monophonic audio signal from a single microphone to reduce background noise. This can be achieved by using many approaches such as spectral subtraction^3,4^, subspace estimation^5^ and gain adjustments based on the short-time signal-to-noise ratio (SNR) estimate^6–9^ and ideal binary mask estimate^10,11^. These traditional single-microphone algorithms are heavily driven by signal processing strategies based on time-averaged signal statistics and certain assumptions about the noise. For example, in spectral subtraction, the noise is assumed to be unmodulated and stationary (as well as broadband and additive), whereas the target speech signal can be retained by preserving the channels with relatively large amplitude modulations. Because of these inherent assumptions, these algorithms tend to perform relatively well only if the background is statistically consistent and predictable, such as a stationary noise. Regarding evaluations of single-microphone techniques,^3^ observed improvement in speech recognition with speech-shaped noise but only moderate improvement with 6-talker babble. CI listeners in^6^’s study showed improvements of 19% on average on speech recognition in speech-weighted noise and small improvement of 7% in 20-talker babble but no significant improvement in a 4-taller babble.^9^ also observed greatest benefit in speech-weighted noise, compared to more realistic street-side city noise and cocktail party noise.

A number of studies evaluated a commercial noise reduction strategy—Advanced Bionic’s ClearVoice^TM^^12^, which works by adjusting the short-term channel gains according to the estimate of instantaneous SNR.^13^ did not see improvements in speech recognition with ClearVoice in steady state speech-spectrum noise. ClearVoice was only shown to increase performance in non-speech noise when combined with other parameter adjustments, such as adjusting maximum comfort levels^14^, or with other technologies such as a remote microphone^15^ and/or multi-microphone noise reduction strategies^16,17^. Similar commercial noise reduction strategies for CIs include the Ambient Noise Reduction (ANR) strategy from^18^ and SNR-NR from Cochlear Limited^19^. Listening with CIs and other hearing assistive devices is especially challenging in scenarios with non-stationary noise, such as multi-talker background interference^20,21^. Therefore, the inherent inability of single-channel noise reduction solutions to effectively remove non-stationary noise interference remains to be the most critical limitation of modern commercial noise reduction strategies for CIs.

Compared to single-microphone algorithms, multi-microphone noise reduction strategies, also commonly referred to as beamforming, involve capturing the audio signals from two relatively closely-spaced microphones and then processing the signals to reduce background noise from a spatial region, typically behind the listener. Beamforming was first introduced to a commercial behind-the-ear CI sound processor by Cochlear Limited in 2005, a decade after it became a standard feature in hearing aid devices^22,23^. Beamforming typically utilizes a front and rear microphone. A sound originating from behind arrives at the rear microphone before the front microphone. This external time delay is used by the signal processing to attenuate the sound from the back. Adaptive beamforming was later introduced to account for moving noise sources that are not always at the back of the listener but could be slightly off to the side^24^. Directionality, the ability to focus on the signal in front of the listener, is typically better with more microphones^25^. However, due to the minimum distance requirement between the microphones and the size of a CI audio processor, it is impractical to have too many physical microphones on a single processor. Binaural beamformers were developed to increase the number of microphone signals available for beamforming by utilizing all four physical microphones from two hearing aids or audio processors for bilateral CI listeners. Reference^26^ showed that a binaural beamformer (4 microphones within 2 hearing aids) provided significantly better benefit in speech-in-noise hearing than monaural directional microphones (2 microphones within 1 hearing aid) regardless of the SNR level. However, The effectiveness of multi-microphone noise reduction strategies is limited to the scenario where the target speech and noise are spatially separated and the listener faces the target all the time. For example,^27^ showed that adaptive binaural beamformer did not show substantial improvement from monaural beamformer when the noise is omni-directional without distinct spatial separation from the target. Additionally, these algorithms can be adversely affected by reverberation, which exists in most listening situations and can degrade source localization^2,9^. The effectiveness of a beamformer is also impacted by how the device is worn by the listener. For a behind-the ear processor, the optimal sitting position of the device on the ear results in the front and rear microphones positioned close to horizontally, which allows for a match between the real delay and the assumed external time delay based on the physical distance between two microphones. This match enables relatively precise cancellation of noise. If the device is worn at an angle, for example, the real time delay of sound traveling from one microphone to the other will be smaller than the delay assumed by the strategy^28^. Considering these restrictions from multi-microphone noise reduction solutions and the limitation of existing single-microphone algorithms in reducing non-stationary noise, we are interested in single-microphone noise reduction strategies that can suppress non-stationary, multi-talker noise interference.

Machine learning, especially deep learning, has transformed many fields, including audio processing. Machine learning appears to have the potential to overcome the limitation of current noise reduction solutions in CIs and hearing aids in reducing non-stationary noise^2^. Research in machine learning-based noise reduction algorithms has increased in the last decade with the advent of more powerful computing resources and the availability of larger datasets. The body of literature on machine-learning based noise reduction has been increasing at a fast pace, in part because researchers evaluate their algorithms with model-based objective evaluation metrics only and then move onto developing the next architecture without behavioral evaluation with human participants^29^. The planning and execution of behavioral experiments, especially those with CI participants, can require complex setups and careful calibration and take much time and resources to complete. Based on the structured review by^30^, there are only 14 studies on machine learning applications for speech and signal processing optimization in CIs (1990–2018). Here, we focus on the work that tested their algorithms on participants with hearing loss, especially CIs. Early work was focused on developing machine learning-based algorithms for suppressing non-stationary, non-speech environmental noise and demonstrated improvement in speech intelligibility among CI listeners^31^. More recent studies have shown improved speech intelligibility in non-stationary background noise such as a multi-talker babble. For example, Ref.^32^ showed improved speech intelligibility in 2-talker (TTB) babble noise among typical hearing (TH) listeners using CI simulations with a deep denoising autoencoder (DDAE) compared to conventional noise-reduction approaches, and further improved the performance among Mandarin-speaking CI listeners when a noise classifier was added to the DDAE algorithm^33^. Reference^34^ demonstrated improved speech intelligibility in 20-talker babble among CI listeners when a neural network algorithm was used to process the noisy speech.

Note that the algorithms mentioned above operate based on *a priori* knowledge of the target and/or background by using the same materials from training during testing. For example, Refs.^31,33,34^ split the same speech corpus into training and testing datasets and used the same noise types to create noisy speech. The algorithm performance is expected to decrease significantly if *unseen* testing data are presented to the algorithms. A larger and more diverse dataset is key to improving algorithm generalizability. Indeed, the performance of algorithms tends to be overestimated when trained and evaluated on congruent data sets or simplified testing scenarios, such as WSJ-2Mix (mixture of 2 utterances from WSJ0 corpus)^35^. Another important aspect of generalizability is algorithm architecture. Recent studies have demonstrated the potential of using recurrent neural network (RNN) algorithms for better generalization by including a classic architecture called long short-term memory (LSTM)^36^. LSTM accumulates information from the past and hence enables the network to form a temporary memory, which is essential for properly managing and learning speech context. Many studies have demonstrated the success of RNN-LSTM based algorithms in speech recognition, enhancement, and separation applications^37–40^. However, the application of such algorithms in noise reduction for hearing aids and CIs has been limited except for a few recent studies. Reference^41^ used an RNN and tested listeners with hearing loss, using the same materials for both training and testing. Reference^42^ and other most recent studies such as^43,44^ addressed both limitations by using algorithms such as LSTM and deep complex convolution transformer network (DCCTN) (all tested with CI listeners) as well as using different materials for training and testing. While the algorithm tested by Ref.^43^ did not result in comparable performance in babble noise to non-speech noise, the RNN from^42^ and DCCTN from^44^ led to a bit more improvement in babble noise than non-speech noise on average.

The present work aims to further explore the potential of machine learning algorithms in reducing non-stationary, multi-talker noise interference. We also aim to develop algorithms with greater generalizability by leveraging a large, custom-created training dataset as well as advanced algorithm architectures, such as RNN. Despite the wide adoption of RNN in modern audio processing systems and in many other domains, RNN architecture suffers from “vanishing gradient” or “short-term memory”. This problem occurs during the training of the network when the gradients diminish as they are backpropagated through time. Consequently, the algorithm may struggle to learn long-term dependencies, impacting tasks like processing lengthy speech instances and capturing information from distant past time steps. Therefore, in addition to RNN, we adopted an architecture known as “Transformer”, which can process input signals all at once through parallel processing, which ultimately leads to a more efficient learning of long-term dependencies^45^. Transformer has gained competitive performance and considerable popularity in speech recognition^46^, speech synthesis^47^, speech enhancement^48^, and audio source separation^45^, as well as other applications such as ChatGpt. We adopted the SepFormer from^45^, a Transformer-based, top-performing algorithm in speech separation applications at the time of this study, according to the Papers with Code website. This state-of-the-art SepFormer algorithm was used here as a reference for the flagship benchmark algorithm to explore the maximum benefit/upper bound of DNN-based noise reduction for CIs, while the RNN algorithm served as a relatively low-complexity, but still advanced algorithm. In addition to including more sophisticated algorithms to assess the potential of DNN solutions and training the algorithms with a large custom-created dataset, the effectiveness of the algorithms was evaluated not only with objective intelligibility measures, but also with CI listeners. Most studies have given priority to intelligibility. However, it is also important to evaluate the perceived quality. In this work, we evaluated both intelligibility and quality to also investigate the impact of the processing algorithms on the subjective quality of the processed speech.

## Methods

### Participants

A total of thirteen adults (seven males) fitted with MED-EL CIs (MED-EL GmbH, Innsbruck, Austria) participated in the study. They were between the ages of 20 and 72 years old (mean = 58.6 years, SD = 14.7 years). The average duration of CI use was 6.5 years (SD = 5 years). Demographic information is detailed in Table 1, including each participant’s default clinical sound coding strategy, which was used when listening to test materials. This study was approved by the Western Institutional Review Board (Protocol 20100066). All experiments were performed in accordance with relevant guidelines and regulations. All participants gave informed written consent prior to testing. Only research participants whose participation in the study would cause financial hardship received financial compensation for their participation.

### Deep neural network architectures

#### RNN

**Figure 1:**
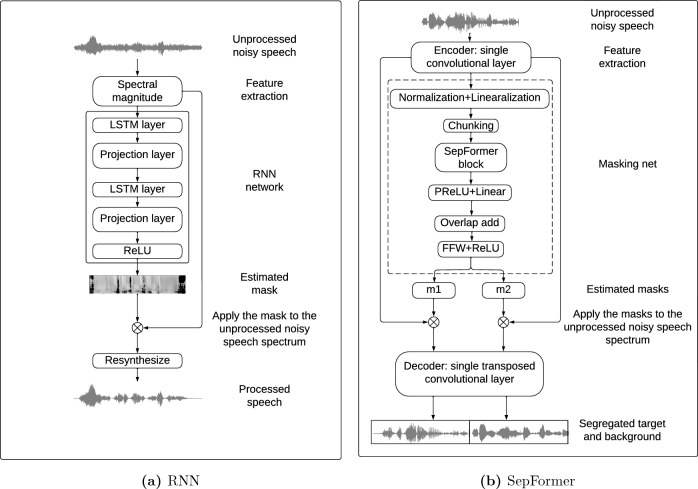
Schematic diagrams of the DNN architecture and signal processing frameworks used in this study: (**a**) RNN and (**b**) SepFormer.

The schematic diagram of the single-channel, RNN-based speech enhancement algorithm is illustrated in Fig. 1a. A clean target speech signal and either speech babble or non-speech noise were mixed to create unprocessed noisy speech. The features used as input to the RNN algorithm were the spectral magnitudes of the short-time Fourier transformation (STFT) of the mixtures (size of Fast Fourier Transform = 512). The spectral magnitudes were extracted using Hamming-windowed frames with a window size of 32 samples and a hop size of 16 samples applied to signals sampled at 16 kHz. “Add-one” log (i.e., adding 1 to the value before taking log) was applied to the spectral magnitude to reduce the influence of values that are smaller than 1. The predicted mask (i.e., the algorithm outcome) was applied to the STFT of the mixture (spectral magnitudes) to generate the “de-noised” spectrum of the mixture and was a continuous “soft” mask (continous gains from 0 to 1, as opposed to a binary mask where the values of the mask are either one or zero). Previous work suggests that “soft” continuous masks result in better speech quality and intelligibility than binary masks under various noise conditions^49,50^. The “de-noised” estimate would ideally consist of the enhanced target speech only. This “de-noised” spectrum was compared with the spectrum of the clean target speech to compute the mean square error (MSE) loss for training optimization. The processed speech estimate was then recovered by resynthesizing the “de-noised” spectrum (i.e., taking the inverse STFT).

The RNN network consisted of an input layer (size = 512) and two hidden LSTM layers (256 units), with each LSTM followed by a projection layer (128 units). A rectified linear unit (ReLU) was used as an activation function. A PyTorch-powered speech toolkit—SpeechBrain—was used to implement, train, and test the RNN algorithm. The Adam optimizer was used for minimizing the MSE loss during the training process^51^, with learning rate set at 0.0001. The algorithm performance was evaluated and monitored with a validation dataset at the end of each learning cycle (i.e., one epoch, containing all training samples). The training was terminated after 100 epochs as the algorithm performance with validation dataset stabilized with no further substantial improvements.

**Table 1:** Demographic information for the participants who participated in this study.

Participant	Gender	Age_T	Age_HL	Dur_CI	#_El	Strategy	Score
ME128	M	70	30	95	10	$\hbox {FS4-p}^{1}$	79
ME149	F	57	27	53	8	$\hbox {FS4}^{2}$	92
ME153	M	71	34	45	12	FS4	91
ME159	F	60	17	64	12	$\hbox {FSP}^{3}$	92
ME185	F	65	8	119	12	FS4-p	76
ME191	M	64	42	40	11	FS4-p	93
ME196	M	65	11	24	11	FS4	92
ME202	M	55	0	40	12	FS4	56
ME203	F	63	5	41	12	FS4-p	73
ME204	M	63	41	33	12	FS4-p	48
ME206	F	72	27	66	12	FS4-p	92
ME207	M	20	0	229	12	FS4	86
SSD100	F	37	24	158	12	FS4-p	68

*Age*_*T* age at the time of testing (years), *Age*_*HL* age at onset of hearing loss (years), *Dur*_*CI* duration of CI use (months), #_*El* number of active electrodes. Strategy: 1—**F**ine **s**tructure processing with *parallel* stimulation in the **4** apical channels; 2—Fine structure processing with *sequential* stimulation in the four apical channels; 3—Fine structure processing with *sequential* stimulation in a variable number of apical channels. Score: IEEE score in quiet (percent correct)^52^.

#### SepFormer

While the light-weight, generic RNN algorithm serves as a proof of concept for DNN algorithms that are more suitable for real-time processing in hearing devices such as CIs, we also implemented the current state-of-the-art algorithm for speech separation applications—SepFormer, to explore the ceiling limit of current DNN technology in noise reduction for CIs (especially non-stationary, multi-talker noise interference). The algorithm architecture is shown in Fig. 1b. Instead of directly feeding STFT features of the noisy mixture to the network as in the RNN algorithm, a single-layer convolutional network was used as an encoder to learn the 2-dimensional features of the input noisy signal (256 convolutional filters with a kernel size of 16 samples and a stride factor of 8 samples). Similarly, at the end of the process, a transposed convolution layer with the same stride and kernel size as in the encoder was used to turn the source-separated features into separate sources. The extracted features of the noisy mixture go into the masking network, which estimates the masks for the foreground (i.e., target speech) and background. These masks were also continuous or soft-decision masks that provided continuous gains from 0 to 1 as in the RNN algorithm. In the masking network, the features were first normalized and processed by a linear layer. They were then buffered into chunks of size 250 along the time axis with an overlap factor of 50%. Next, they were fed into the core of the masking net—SepFormer block. This block consists of two transformer structures that learned both short and long-term dependencies. More details about this algorithm can be found in^45^. The output of the SepFormer block was then processed by a parametric rectified linear unit (PReLU) and linear layer. The overlap-add scheme, described in^53^, was used to sum up the chunks. This summed representation was passed through two feed-forward layers and a ReLU activation function to finally generate the masks for both the foreground and background sources (m1 and m2 in Fig. 1b). The training procedure and infrastructure were the same as for the RNN algorithm. Note that both RNN (bi-directional LSTM) and SepFormer used non-causal processing with access to future frames.

### Dataset

#### Training and validation datasets

Clean target speech audio signals were generated from the LibriSpeech ASR corpus, an open-source, large-scale ($\sim$1000 h, 2484 speakers) corpus of read English speech. The speech audio samples were extracted from read audiobooks from the LibriVox initiative, and every recording was carefully segmented and aligned with the text transcript. This study used the training subset that contains 100 h of clean speech. The non-speech materials were from WHAM!, an open-source, large-scale ($\sim$ 82 h) dataset of noise audio signals. The noise data samples were recorded from different urban spots across the San Francisco Bay Area, mainly comprising restaurants, cafes, bars, and parks. Each recording was processed to eliminate any parts that include intelligible speech. Four types of noisy speech were created for training: target in non-speech noise (WHAM! noise), and target in 1, 2, 4-talker babble. Recordings from LibriSpeech were mixed to form multi-talker babble. All speakers in the mixture are distinct from one another and speaking different content. Each type of noisy speech was mixed at SNRs from 1 to 10 dB in 1-dB steps, with equal representation. The loudness of target was kept constant at ITU level of 29. A total of 5590 speech-in-noise mixtures were created for each noise type, which resulted in a training dataset of $\sim$ 30 h. The validation dataset contains 410 speech-in-noise mixtures for each type of noisy speech. Note that, as for training optimization, an MSE loss function was used to evaluate the algorithm performance during validation step. To speed up the process of algorithm preparation, the training and validation were conducted on a graphics processing unit (GPU, Google Colaboratory).

#### Testing dataset

Clean target speech materials were extracted from the IEEE corpus, which is often used in auditory research and clinics. This corpus contains recordings of 33 different talkers. Most speakers read the full set of 720 IEEE sentences. Three hundred and forty sentences from the IEEE corpus (target sentences) were mixed with CCITT noise (speech shaped stationary noise according to ITU-T Rec. G.227) or TTB babble at SNRs of +1, +5 and +10 dB. Two-talker babble mixtures were produced by mixing the IEEE recordings of 120 sentences that were not chosen as target sentences. Male recordings were used for target sentences while female recordings were used to generate TTB babble, and the two were combined to create the noisy mixtures. Note that the IEEE corpus was never used during training or validating the algorithms. The non-speech noise used for training and validation was relatively sparse environmental sounds, whereas the CCITT noise used for testing was steady-state, speech-shaped noise. This testing dataset was used for both objective evaluations and testing CI participants (procedures described below in “Objective evaluations” and “Behavioral testing”, respectively). For each of the three types of objective evaluation measures (as detailed in “Objective evaluations” below), all 340 target sentences were used for each condition (e.g., 1-dB SNR, TTB noise, SepFormer). For testing with CI participants, a total of 260 sentences [20 sentences * (3 processing conditions * 2 noise types * 2 SNRs + quiet) = 20 * 13 test conditions = 260 sentences] were randomly selected from the pool of 340 target sentences. Note that objective evaluations were only carried out for the testing, not for the validation process.

In addition to testing speech intelligibility scores, we also asked CI participants to evaluate the quality of the de-noised speech mixtures with CCITT and TTB noise types, under RNN and SepFormer processing conditions. We asked CI participants to evaluate the quality of the clean speech in quiet (using the remaining 240 IEEE sentences that were not used for target sentences or forming TTB babble), because the algorithms removed the noise interference to the extent that the de-noised speech mixtures sounded noise-free in most cases. Clean speech measures were included to account for the fact that, even if the algorithm perfectly restored the original signal, CI users do not achieve 100 percent correct scores in quiet. Looking at performance in noise, without understanding the upper bound of performance can be misleading. If there were enough time and resources available, evaluating unprocessed noisy speech could have provided another valuable point of reference. But in the meantime, we were concerned that including the unprocessed noisy mixture would introduce a floor in the scaling procedure that would increase and compress ratings of algorithm outputs, compromising the sensitivity of the procedure.The algorithm testing was conducted on a MacBook Pro, i7 core (2.2 GHz) central processing unit (CPU).

### Testing procedures

#### Objective evaluations

The DNN algorithms were first evaluated quantitatively using three commonly used acoustic evaluation metrics: scale-invariant source-to-distortion ratio (SI-SDR)^54^, short-time objective intelligibility (STOI)^55,56^, and “perceptual” evaluation of speech quality (PESQ)^57,58^. These objective evaluation measures helped inform the overall expected benefit before conducting behavioral listening tests with CI users. All three evaluation metrics compare the clean reference speech and the same speech recovered from the noisy mixture and quantify the agreement between the two, which allows for an estimate of the improvement in quality and intelligibility due to algorithm processing when compared to the metric for the unprocessed noisy mixture.

The traditional SDR metric decomposes the estimated source into four components representing respectively the true source, spatial distortions, interference, and artifacts. The final SDR score is computed by calculating the ratio of the source energy to the sum of all other projection energies (i.e., spatial distortions, interference, and artifacts) as described in^59^. The SI-SDR with slight modifications as described in^54^ has been shown to be more robust and is now the standard for DNN noise reduction evaluation.

The STOI metric was initially designed to predict the intelligibility of speech processed by enhancement algorithms.^60^ demonstrated that STOI outperformed all other measures for predicting intelligibility of CI listeners. The STOI first applies time-frequency analysis to both clean reference and processed speech signals. An intermediate intelligibility measure is obtained by estimating the linear correlation coefficient between clean and processed time-frequency units. The final STOI score is the average of all intermediate intelligibility estimates from all time-frequency units.

The PESQ score ranges between − 0.5 and 4.5. It was calculated by comparing the reference signal with the processed signal by deploying a perceptual model of the human auditory system. The PESQ is computed as a linear combination of average disturbance value and average asymmetric disturbance value. The parameters for the linear combination can be further modified towards predicting different aspects of speech quality. More details can be found in^57,58^ and^61^. In general, the PESQ has been shown to be capable of reliably predicting the quality of processed speech.^50^ showed that parameter optimization for reverberation suppression algorithms based on the PESQ metric resulted in better performance than the STOI metric. Therefore, in the present context, the PESQ was chosen to detect and quantify the overall effects of DNN processing on the signal quality.

#### Behavioral testing

***Test setup***. All participants were tested using their everyday clinical program, as listed in Table 1. The stimuli were delivered to each participant’s own audio processor through a direct audio input (DAI) cable, which attenuates the microphone inputs by approximately 30 dB relative to the direct input signal. The DAI also bypasses the front-end microphone directionality (i.e., beamformer) and wind-noise reduction features. The test stimuli were presented at an input level corresponding to 65 dB SPL (root mean square (RMS) level). None of the participating participants used MED-EL’s channel-specific, ambient and transient noise reduction algorithms in their daily maps at the time of testing. At the beginning of the testing session, the moderator provided instructions regarding the study procedures, and then connected the recipient’s processor to the audio port of a Windows-based touchscreen tablet (Microsoft Surface Pro) through the DAI cable. The proprietary psychophysical software suite, PsyWorks v.6.1 (MED-EL GmbH, Innsbruck, Austria), was used to present the speech stimuli from the tablet to the audio processor. The calibration was performed using a built-in feature within the PsyWorks software and was adjusted according to each recipient’s audio processor type.

***Intelligibility and quality measurements***. Each processing condition (unprocessed, RNN, SepFormer) was evaluated with a list of twenty sentences from the testing dataset for each combination of masker type and SNR (+10 and +5 dB SNR). Each participant performed a total of 13 tests (2 masker types $\times$ 3 processing conditions $\times$ 2 SNRs + 1 quiet). The testing was carried out in a self-administered manner. The participants used the tablet and PsyWorks to present the speech materials to their own audio processors. Participants were assigned a unique presentation order using a Latin square design and were blinded to the processing condition. The participants either vocalized their responses through a microphone located in front of them or typed them, according to their preference. Spoken responses were captured in real-time by an automatic speech-to-text module (Google API). Spoken responses could be edited by typing before submission, and the PsyWorks software automatically scored words as correctly or incorrectly identified. Words containing additions, substitutions, or omissions were scored as incorrect. The percent correct scores for each condition were calculated by dividing the number of correct words by the total number of words. After each list, the percent correct was displayed and stored electronically. All participants were native English speakers, and none of the participants that elected to speak their responses had speech difficulties that jeopardized the scoring of their responses. We also asked participants to rate the quality of the speech processed by both algorithms, in both masker types, as well as the unprocessed clean speech (i.e., quiet). Subjective evaluation scores, according to the mean opinion score (MOS) scale, assign a numerical measure of the human-judged overall quality: 1 corresponds to “bad”, while 5 represents “excellent”. The total testing time for all experimental conditions tested was approximately 2.5 h including multiple breaks.

### Statistical analyses

Statistical analysis was performed with RStudio (R version 4.3.1). A series of linear mixed effects models of percent correct scores (lmer function, lme4 package in RStudio) with different combinations of the fixed effects (i.e., SNR, processing condition, and masker type) and with participant intercept being a random effect, were fitted and compared based on Akaike information criterion (AIC, aictab function, MASS package). A linear mixed effect model was used to model the rated quality of speech with processing condition and masker type, and their interactions as fixed effects, with random effects to account for the variability associated with participant IDs. The “anova” function from the car package was employed to compute the type-III sequential sum of squares, assessing the predictive impact of independent factors and their interactions in the best fitted model. We used estimated marginal means (EMMs, emmeans package in R, version 1.8.9) to estimate the expected value of the percent correct scores from behavioral testing in each condition (e.g., CCITT, 5-dB SNR, unprocessed). It is a way to understand the expected value of the dependent variable (i.e., percent correct scores in this case) while accounting for the effects of other variables (noise types, SNRs, processing conditions). This is commonly used in regression analysis and ANOVA (analysis of variance) to interpret the impact of independent variables on the dependent variable. Although the data from objective evaluations reasonably follow normal distribution (via histogram inspection), they did not pass a more conservative test for normality such as the one-sample Kolmogorov-Smirnov test. Therefore, we adopted the non-parametric alternative to the paired t-test, the Wilcoxon Signed-Rank test.

### Ethics approval

This study was approved by the Western Institutional Review Board (Protocol 20100066). All participants gave informed written consent prior to testing. Research participants whose participation in the study would have caused financial hardship received financial compensation for their participation.

## Results

### Objective evaluations

**Figure 2:**
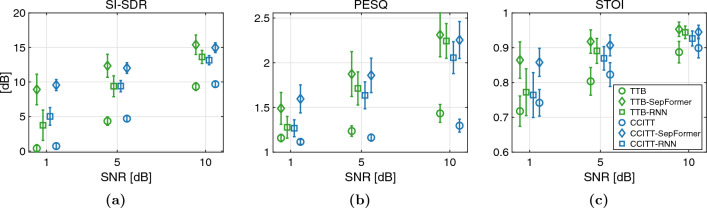
(**a**–**c**) Objective evaluation scores for unprocessed baseline and signals processed with DNN algorithms. Higher scores are better. Statistical significance was found for all cases between the processed and unprocessed conditions, as well as between two processed conditions. *SI-SDR* scale-invariant signal to distortion ratio, *PESQ* perceptual evaluation of speech quality, *STOI* short-time objective intelligibility, *TTB* two-talker babble, *CCITT* speech shaped noise, *SNR* signal to noise ratio. Note that for PESQ scores were mapped to MOS (mean opinion score), which is a numerical measure of the human-judged overall quality of an event or experience^65,66^. STOI scores are expected to have a monotonic relation with the subjective speech-intelligibility, where a higher score denotes more intelligible speech^55,56^.

Three objective metrics (SI-SDR, PESQ, STOI) were evaluated for three processing conditions (“unprocessed”, “processed by RNN”, and “processed by SepFormer”) and two masker types (TTB and CCITT). The algorithms produced significant improvements across all objective evaluation measures. The objective evaluation scores for both RNN and SepFormer algorithms with 340 mixtures (see “Testing dataset”) are shown in Fig. 2a–c. As shown in Fig. 2a, RNN processing significantly improved SI-SDR scores over the unprocessed condition across all SNRs, for both masker types (TTB and CCITT maskers, Wilcoxon Signed-Rank tests, $p < 0.0001$). The algorithms produced significant improvements in the other two objective metrics as well: PESQ (Fig. 2b) and STOI (Fig. 2c). These improvements are also statistically significant (Wilcoxon Signed-Rank tests, $p < 0.0001$). Although statistically significant, the improvement in the speech intelligibility metric (i.e., STOI) was not as large as in the two speech quality metrics (i.e., SI-SDR and PESQ). This is probably due to ceiling effects: the SNR tested was high overall (minimally 1 dB SNR) and the predicted speech intelligibility was high overall in these test conditions^62^. These STOI values are similar to what was reported for a STFT-based algorithm tested at 0 dB SNR^63^. For the SepFormer algorithm, the objective evaluation scores for the processed audio signals had even more separation from the unprocessed baseline, indicating better performance by SepFormer than RNN (Wilcoxon Signed-Rank tests, $p < 0.0001$ for all three metrics, across all SNRs, and for both masker types). The superior performance of SepFormer over RNN is especially evident in the noisier, 1-dB SNR condition across all three evaluation metrics. These Wilcoxon Signed-Rank tests survived multiple comparisons (54 comparisons) using false discovery rate procedures^64^.

### Behavioral testing with CI listeners

#### Speech-in-noise intelligibility

The speech intelligibility scores were measured behaviorally as percent correct for the following three processing conditions: “unprocessed”, “processed by RNN”, and “processed by SepFormer”. As with the objective evaluation measures, CI listeners were tested with both TTB and CCITT maskers. The speech intelligibility performance was measured at both 5- and 10-dB SNR test conditions. The DNN algorithms introduced improvements in speech intelligibility scores over the unprocessed condition at both SNRs and with both masker types for almost all CI listeners tested (only a few exceptions occurred mostly with RNN processing when the masker type was CCITT). A model was created by modeling the percent scores with processing condition, SNR, masker type, and the interaction between processing condition and masker type as fixed effects, with random effects to account for the variability associated with participant IDs: $model = lmer(percent\_correct \sim processing\_condition + SNR + masker\_type + processing\_condition:masker\_type + 1|participant)$. There was a significant main effect of SNR ($F[1, 143] = 135.75, p < 0.0001$), processing condition ($F[2, 143] = 113.96, p < 0.0001$), masker type ($F[1, 143] = 10.49, p = 0.0015$), as well as interaction between processing condition and masker type ($F[2, 143] = 3.81, p = 0.025$). The homoscedasticity assumption of the model was checked and validated by plotting the model residuals against the fitted values, and the sample Quantiles against theoretical Quantiles.

**Figure 3:**
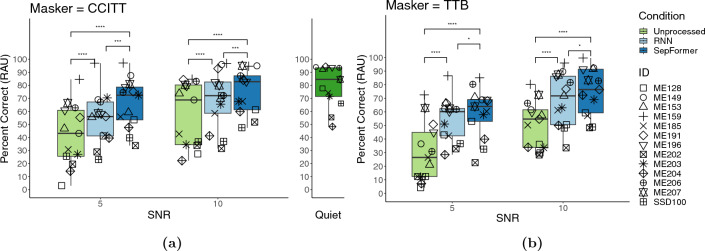
Individual sentence recognition performance (referenced to performance in quiet) plotted as a function of each processing condition for both SNR conditions, separately CCITT and TTB maskers. The boxes depict the values between the 25th and 75th percentiles, and the whiskers represent minimum and maximum values. Medians are shown as horizontal lines. Significance stars: $$0.05>*\ge .01$$, $$0.001>***\ge 0.0001$$, $$0.0001>****$$.

Both the RNN and SepFormer algorithms improved speech intelligibility for both non-speech (Fig. 3, left—CCITT) and speech (Figure 3, right—TTB) masker types, across both SNR conditions, with their performance approaching to the levels in quiet. Estimated marginal means (EMMs) with standard errors and confidence intervals for the three processing conditions within each Masker Type and SNR condition are shown in Table 2.

With CCITT masker, post-hoc pairwise comparisons revealed that the scores from RNN (EMM difference (diff) = 11.94%, $p <0.0001$) and SepFormer (diff = 20.91%, $p <0.0001$) processing condition were significantly higher than those from unprocessed conditions under the 5-dB SNR configuration. SepFormer also outperformed RNN by 8.97% on average ($p =0.0005$). Although the baseline unprocessed condition improved when the SNR was increased to 10 dB (diff = 15.3%, $p <0.0001$), both RNN and SepFormer continued to improve speech intelligibility scores, with SepFormer still outperforming RNN (diff = 8.97%, $p = 0.0005$).

The aforementioned benefits from RNN and SepFormer processing over unprocessed conditions, with SepFormer outperforming RNN, are also evident with TTB masker type. However, TTB masker worsened speech intelligibility significantly for the baseline unprocessed condition (diff = 8.99%, $p = 0.0002$), reflecting more deleterious impact from speech noise on speech intelligibility than non-speech noise, and still both RNN and SepFormer algorithms improved the speech intelligibility to similar levels as those achieved in CCITT-masker conditions. Unlike the significant effect of masker type on performance with the baseline unprocessed condition, there was no significant difference in speech intelligibility score between TTB and CCITT conditions when measured using the two algorithms (RNN: diff (CCITT-TTB) = 0.24%, $p = 0.92$; SepFormer: diff (CCITT - TTB) = 3.5%, $p = 0.13$). In other words, both RNN and SepFormer algorithms introduced *greater* speech enhancement on average when the masker was *speech* as opposed to when it was stationary, non-speech noise.

**Table 2:** EMMs for processing conditions with different masker type and SNR configurations.

Masker type	SNR	Processing condition	EMMs	SE	95% CI
CCITT	5	Unprocessed	42.1	5.32	[30.8, 53.3]
CCITT	5	RNN	54.0	5.32	[42.8, 65.2]
CCITT	5	SepFormer	63.0	5.32	[51.8, 74.2]
CCITT	10	Unprocessed	57.3	5.32	[46.1, 68.5]
CCITT	10	RNN	69.3	5.32	[58.0, 80.5]
CCITT	10	SepFormer	78.2	5.32	[67.0, 89.4]
TTB	5	Unprocessed	33.1	5.32	[21.9, 44.3]
TTB	5	RNN	53.8	5.32	[42.5, 65.0]
TTB	5	SepFormer	59.5	5.32	[48.3, 70.7]
TTB	10	Unprocessed	48.3	5.32	[37.1, 59.5]
TTB	10	RNN	69.0	5.32	[57.8, 80.2]
TTB	10	SepFormer	74.7	5.32	[63.5, 86.0]

*SE* standard error, *CI* confidence interval.

***Comparison with other studies***

**Figure 4:**
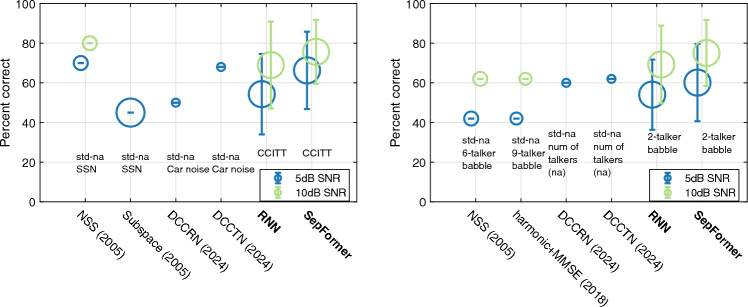
Comparison of algorithm performance between current (RNN, SepFormer) and previous studies^3,5,44,67^ tested in similar SNR and noise-type conditions with CI participants. The size of the circle is proportional to the number of CI participants tested in the study. The center of the circles align with population mean and the error bar is 1 standard deviation. Note that values from some studies are visual approximation from the figures, which explains the lack of error bar (indicated by text annotation: “std-na”). NSS, Subspace are traditional signal processing algorithms, while harmonic+MMSE is a statistical based algorithm that has neural network structure. DCCRN, DCCTN, RNN, SepFormer are deep-learning based algorithms. *NSS* nonlinear spectral subtraction, *MMSE* minimum mean square error, *DCCRN* deep complex convolutional recurrent network (CNN + RNN), *DCCTN* deep complex convolution transformer network, *SSN* speech-shaped noise.

Figure 4 shows the comparison of percent correct improvements introduced by the algorithms in current study and those from several previous studies. In non-speech stationary noise (Fig. 4, left), algorithms from current studies achieved comparable improvements in intelligibility to traditional, signal processing algorithms^3,6,8^. The benefit from those traditional signal processing algorithms is limited in speech babble noise (Fig. 4, right;^3^ – 6-talker babble;^6^ – 4-talker babble). However, both RNN and SepFormer algorithms introduced even *more* improvements in a TTB babble noise compared to steady-state noise, indicating a substantial advantage of deep-learning based noise reduction algorithms in reducing non-stationary, multi-talker noise interference.

#### Subjective evaluation of the speech quality

**Figure 5:**
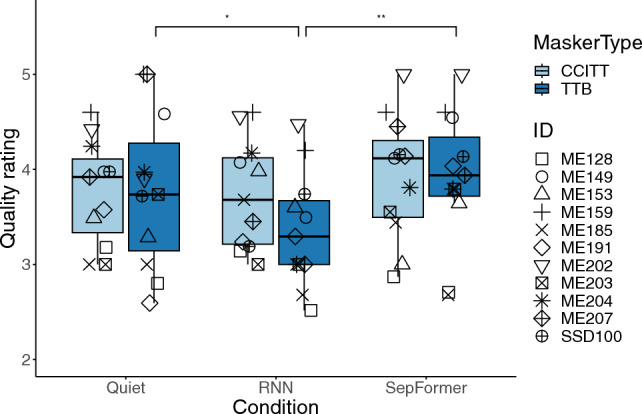
Two blocks of testing were conducted for the “Quiet” condition to equalize the number of samples since there were two types of maskers for each processing condition. Note that the quality was evaluated for two processing conditions in 10-dB SNR. Due to time restriction, the quality was not evaluated for 5-dB or unprocessed conditions. Significance stars: $$.05>*\ge 0.01$$, $$0.01>**\ge 0.001$$.

In addition to completing speech intelligibility tests and as shown in Fig. 5, participants rated the quality of the speech processed by both RNN and SepFormer algorithms in both TTB and CCITT maskers as similar to that of the unprocessed signals before mixing with noise (i.e., in quiet). A linear mixed effect model was carried out, modeling the rated quality of speech with processing condition (quite, RNN, SepFormer) and the type of masker (TTB, CCITT), and the interaction between processing condition and masker type as fixed effects, with random effects to account for the variability associated with participant IDs: $model = lmer(quality \sim processing\_condition * masker\_type + 1|participant)$. The test results indicated that there was a statistically significant effects from the processing condition ($F[2, 55] = 4.99, p = 0.01$), but not from the type of masker ($F[1, 55] = 1.78, p = 0.19$), or two-way interaction between the processing condition and the type of masker ($F[2, 55] = 1.73, p = 0.19$). With TTB masker, the participants rated Quiet condition slightly higher than RNN by 0.42 ($p = 0.045$), and SepFormer slightly higher than RNN by 0.53 ($p = 0.008$). No other statistically significant pairwise comparisons were found [TTB: Quiet-SepFormer (diff = − 0.12, $p = 0.78$); CCITT: Quiet-RNN (diff = 0.027, $p = 0.99$), Quiet-SepFormer (diff = − 0.16, $p = 0.62$), RNN-SepFormer (diff = − 0.19, $p = 0.52$)]. This suggests that algorithm processing overall did not significantly distort the speech quality while suppressing the background interference.

#### Improvement with algorithms vs. demographic factors

We performed a linear mixed effects model with the change in score relative to the unprocessed noisy condition as the dependent variable, masker type (TTB/CCITT), algorithm (RNN/SepFormer), and demographic factors as independent variables, and ID as a random factor. The demographic information is listed in Table 1, including gender, age at testing, duration of CI use, age at onset of hearing loss, number of active electrodes, and coding strategy. A stepwise regression analysis was performed on the full linear model, and the output of the stepwise analysis was a linear model that only included masker type and DNN algorithm, with ID as a random factor. Demographic factors always increased the Akaiki criterion of the linear model. This suggests that the potential benefit of the DNN-based speech enhancement algorithms that has been shown so far was not related to any of the selected demographic factors. The data sample might not be powered sufficiently to establish relationships between benefits from algorithms and demographic factors. However, considering the large variability that is typical of CI population, these results, for this sample, demonstrate the promise of DNN algorithms towards clinical application in the near future for better noise reduction in more complex listening environments (e.g., with multi-talker noise interference).

## Discussion

In this work, we implemented, trained, and tested the RNN and SepFormer algorithms for noise reduction in CIs using a training dataset with custom configurations (or mixing). The algorithms were evaluated with objective evaluation measures and behaviorally with a total of 13 CI listeners. Compared to widely adopted classification and regression algorithms such as convolutional neural network (CNN) algorithms, RNN does not require an input of fixed dimensions such as an image file with standardized size, which makes it more suitable for processing audio samples of varying lengths. RNN also keeps a memory of prior information and makes predictions based on both previous and current information (i.e., through LSTM). This feature is important for input signals such as speech, of which segments of information across time are not independent but rather interconnected with each other. SepFormer is a transformer-based algorithm that takes the step further by extending RNN’s sequential processing to parallel processing, which best suits the need for processing longer sequences of signals. These two algorithms were then trained with a large custom dataset containing various target-noise configurations, totaling 22,360 instances for each training iteration. More specifically, we created the large training and validation dataset from commonly used speech and non-speech corpora in machine-listening field—LibriSpeech and WHAM!, respectively. The IEEE corpus, which is more common in hearing research, and CCITT noise were used for creating the testing dataset. The algorithms were also trained more extensively with 100 learning cycles (epochs), to explore the full capacity of the algorithms in noise reduction performance.

Both RNN and SepFormer algorithms significantly improved CI listener’s speech intelligibility in noise relative to unprocessed, noisy speech (almost to the levels in quiet in certain conditions). The improvement in speech intelligibility in stationary non-speech noise was comparable to those gains achieved by traditional signal processing strategies. More importantly, both RNN and SepFormer introduced substantially ***more*** improvements in non-stationary ***multi-talker*** noise interference, where conventional signal processing strategies are limited, than in stationary noise. Similar to our study,^42^ also used RNN and tested speech reception thresholds at 50% correct in both TTB and non-speech traffic noise. They also showed more improvement in TTB than traffic noise.

The objective evaluation measures did not predict the observed advantage of the two DNN algorithms in multi-talker background noise (i.e., more improvements in multi-talker background noise than in non-speech, CCITT noise), which underscores the importance of testing the algorithms with CI participants. It might be due to the fact that these objective evaluation measures were developed based on data from TH listeners. In this study, these metrics were calculated to mainly inform rather than replace the participant testing. Even the metrics adapted for CI listening can never fully approximate the actual testing with CI listeners due to the many sources of variability in CI outcomes^68,69^. It is also noteworthy that the improvements in intelligibility did not come at the price of compromised sound quality of the speech overall among CI listeners. The fact that there were no significant contributions from any demographic disparity across participants suggests that the algorithms have the potential to broadly benefit the general CI population regardless of their hearing etiology, duration of deafness, experience with CI listening, and processor settings; although, it should be noted that the predictive power of the model was limited by the number of participants. Nevertheless, these results show the promise of using machine-learning based algorithms as a complementary or even replacement algorithm for current existing signal processing strategies, to tackle more complex listening challenges involving multi-talker noise interference.

Given that SepFormer was the top-performing, state-of-the-art algorithm for speech separation and enhancement at the time of the study, while RNN is a basic two-layer template algorithm, it is not surprising that the SepFormer outperformed RNN in every scenario. It provided an estimate of the current upper limit to which a DNN-based strategy could restore speech comprehension when paired with CI devices. However, SepFormer is a very complicated algorithm, containing over 26 million parameters. The processing time of such a computationally heavy algorithm turned out to be almost 5 times the duration of the incoming signal on average, which renders it not suitable for a real-time applications such as a CI audio processor. Note that the inference and calculation of the processing time was conducted on CPU (MacBook Pro i7 2.2GHz) instead of a dedicated GPU. The time constraint for the processing delay in a real-time device such as CI should be below about 10–20 ms to avoid disturbance in speech production and audio-visual integration^42,70–72^. This timing delay should be even lower for individuals with single sided deafness who are fitted with CI on the side with hearing deprivation while receiving acoustic input on the other side^73^. Another limitation comes from the high demand for computational power and memory. Thus, at this time, it is probably unrealistic to execute these highly complex state-of-the-art algorithms even in high-end audio DSPs (digital signal processors) used in CI processors. The RNN algorithm, on the other hand, is a much computationally lighter algorithm and the processing only took 3% of the input signal duration on average. This implies that RNNs can in theory retain low-latency inference, which makes them better-suited DNN algorithm for real-time speech enhancement in CIs. Note that both RNN and SepFormer used non-causal processing, with access to future frames. The next step would be to extend the models to causal processing to stay within the tolerable range of delays. At the time of manuscript revision,^74^ published a modified version of SepFormer to account for causal processing. A uni-directional LSTM can be used in the RNN to consider causal processing. At the time of this study, Oticon launched the world’s first hearing aid that comes embedded with a new platform capable of supporting on–board DNN processing—Oticon More. This development is encouraging for those in the hearing space who are developing DNN-based noise-reduction solutions for CIs.

One limitation of this work is that the algorithms were trained and evaluated solely in fairly low albeit positive SNRs. Negative SNRs, particularly in scenarios with multi-talker interference, pose unique challenges that merit further investigation. An immediate future step would be to expand the training and testing to negative SNRs. However, as shown by^75^, there were very few situations that had recording of negative or even close to 0-dB SNRs. The average SNR was approximately 5 dB for speech-in-babble noise. Another expansion on the training and testing materials would be to include reverberation, which is a common source of signal degradation.^76^ tested DNN algorithms with CI participants in reverberant noisy listening conditions and showed that in such conditions, the algorithms needed to have access to more than one microphone to achieve significant improvement. Reference^27^ reported that multi-microphone noise reduction solutions could improve speech intelligibility across all noise types tested, while the single-microphone, STFT-based algorithm led to smaller benefit. This advantage from multi-microphone solutions highlights the fact that the DNN-based single-microphone algorithms can work with multi-microphone solutions for even greater outcomes in more challenging listening situations. As bilateral CI implantation is increasingly becoming a standard of care, bilateral DNN solutions (e.g.,^77^) should also be considered in the future. Multi-microphone settings could also help create positive SNRs (via beamforming) where the single-microphone algorithms are more effective. Other future plans include optimizing the aforementioned DNN algorithms and testing them in real-time in research processors with CI listeners. The training and testing should also be expanded to languages other than English. Finally, the deployment of the DNN algorithms in research processors should be tested against existing commercial solutions for noise reduction.

## Data Availability

The LibriSpeech ASR corpus, WHAM!, and IEEE datasets are publicly available at no cost. The customization scripts have been made publicly available (see below under “Code Availability”). The de-identified behavioral data with CI participants have been made publicly available along with the data analyses code (see below under “Code Availability”).

## Code availability

Code for model implementation, training, evaluation, code for dataset preparation, code for objective evaluation and data analyses have been made publicly available on Open Science Framework.
